# Intermittent Suckling in Combination with an Older Weaning Age Improves Growth, Feed Intake and Aspects of Gastrointestinal Tract Carbohydrate Absorption in Pigs after Weaning †

**DOI:** 10.3390/ani6110066

**Published:** 2016-10-25

**Authors:** Diana L. Turpin, Pieter Langendijk, Tai-Yuan Chen, John R. Pluske

**Affiliations:** 1 School of Veterinary and Life Sciences, Murdoch University, Murdoch, WA 6150, Australia; J.Pluske@murdoch.edu.au; 2 South Australian Research and Development Institute, Roseworthy Campus, JS Davis Building, Roseworthy, SA 5371, Australia; Pieter.Langendijk@trouwnutrition.com (P.L.); edgecty@gmail.com (T.-Y.C.)

**Keywords:** pig, intermittent suckling, weaning, intestine

## Abstract

**Simple Summary:**

The weaning of piglets involves abrupt changes that often cause reduced production and predisposition to gastrointestinal tract dysfunction. Increasing the weaning age or using gradual weaning are techniques that can improve post-weaning performance. In this study, it is shown that an older weaning age (day 35) in combination with intermittent suckling (daily separation of piglets from the sow for 8 h in the week before weaning) improved growth rate, feed intake and aspects of gastrointestinal tract carbohydrate absorption after weaning. However, intermittent suckling and weaning at 28 days did not seem to improve post-weaning performance in this study.

**Abstract:**

This study tested the hypothesis that intermittent suckling (IS) with or without an older weaning age would improve post-weaning gastrointestinal tract (GIT) carbohydrate absorptive capacity in pigs while reducing post-weaning stress and aspects of the inflammatory response. Three weaning regimes using primiparous sows were compared: (1) conventional weaning (CW28) (*n* = 22), where piglets were weaned conventionally at day 28; (2) IS28 (*n* = 21), where IS started at day 21 until weaning at day 28; and (3) IS35 (*n* = 21), where IS started at day 28 until weaning at day 35. Sugar absorption tests (10% mannitol or 10% galactose) were used to measure GIT absorptive capacity. All measured parameters were compared in relation to weaning across treatments (i.e., different physiological ages were compared). The IS35 pigs grew fastest in the 12 days after weaning (*p* < 0.01) and had the highest solid feed intake before and after weaning (*p* < 0.05). Irrespective of treatment, pre-weaning mannitol levels were higher than post-weaning levels (*p* < 0.01), whereas post-weaning galactose levels were highest in IS35 pigs (*p <* 0.01). Cytokine data did not show any treatment effects. In conclusion, these data suggest that IS in combination with an older weaning age (day 35) improved post-weaning adaptation as evidenced by improvements in performance measures and galactose absorption. However, IS28 did not improve post-weaning performance.

## 1. Introduction

Weaning under commercial conditions at three to four weeks of age is a stressful and abrupt process, with pigs typically having difficulty adapting to the sudden dietary, environmental and social changes involved. Consequently, their level of feed intake in the immediate post-weaning period is usually insufficient to meet maintenance energy requirements [1]. Inadequate feed intake also contributes to intestinal inflammation [2], thereby compromising gastrointestinal tract (GIT) structure and function, predisposing piglets to enteric disease such as post-weaning collibacillosis [3]. Furthermore, stress associated with the process of weaning compromises GIT barrier function, mediated in part by the upregulation of receptors for corticotrophin releasing hormone (CRH) [4]. Collectively, these processes cause a post-weaning ‘growth check’ and contribute to poorer production efficiency and increased mortality and (or) morbidity. This effect appears to be more marked in progeny from primiparous sows as evidenced by reduced growth rates, a higher acute phase protein response and higher mortality rates than sow progeny during the nursery period (day 28 to 60) [5].

Increasing weaning age (five to seven weeks) improves GIT structure [6], increases post-weaning feed intake [7] and reduces stress [7]. However, an extended lactation causes fewer litters per sow per year and may impact production in the breeding herd [8]. Intermittent suckling (IS) is a process whereby piglets are separated from the sow for a specified period of time each day in the latter stage of lactation, and has the potential to uncouple sow reproductive performance from weaning age. A reduction in suckling stimulus increases the percentage of sows ovulating [9], which, in turn, may permit mating during lactation [10,11]. By mimicking the increasing amount of time the sow would naturally spend away from her piglets, IS also has the potential to increase solid feed intake in piglets before weaning, resulting in improved dry feed intake and growth after weaning [9,12]. Additionally, it has been suggested that short periods of separation from the sow with [13] or without [14] fasting before weaning could induce some essential adaptive changes (structural and functional) in the GIT, resulting in quicker maturation, partly protecting piglets from some of the adverse effects of weaning. A study by Berkeveld et al. [15], in which piglets were subjected to IS for seven days before weaning, reported favourable post-weaning outcomes with respect to small intestinal (SI) morphology, which were improved even more when IS was combined with an extended lactation (day 33 versus day 26). However, pre-weaning GIT adaptive changes were not examined in the study by Berkeveld et al. [15], and neither were stress and inflammatory responses.

The hypothesis tested in this study was that piglets subjected to IS would have improved post-weaning performance, GIT absorptive capacity of selected carbohydrates and a reduced inflammatory and stress response, with minimal effect on pre-weaning performance compared to piglets from a conventional weaning (CW) regimen. It was anticipated that extending lactation by one more week in IS piglets (weaning at day 35 versus day 28) would improve post-weaning performance and GIT absorptive capacity to a greater extent. Progeny from primiparous sows was used, as there was limited data on their responses to IS regimens.

## 2. Materials and Methods

The experimental design and procedures were approved by the Animal Ethics Committees of Primary Industries and Resources South Australia (07/12) and Murdoch University (N2553/13).

### 2.1. Animals, Housing and Diet

The experiment was a completely randomised block design conducted in six replicates between April and October 2013 at the Pig and Poultry Production Institute (PPPI) (Roseworthy Campus, University of Adelaide, Roseworthy, Australia). Sixty-four primiparous sows (Large White and Large White Landrace terminal line) and their litters were used in six batches consisting of 9, 11, 11, 10, 11 and 12 sows. Sows were mated at the experimental farm and group-housed during gestation. A week before farrowing, sows were moved into individual farrowing crates (2.1 m × 0.6 m) within farrowing pens (2.1 m × 1.92 m). Once born, litters were provided with an infrared lamp until day 10, and floor heating until weaning. Artificial lighting was provided between 08:00 and 17:00. Litter size varied at birth from 3 to 15 live piglets and was standardised within three days of farrowing by cross fostering within each batch, resulting in an average of 11 piglets per sow. Within 24 h of farrowing, piglets received a 1 mL intramuscular (IM) iron injection (Feron 200 + B12, 200 mg/mL iron dextran and 40 μg/mL cyanocobalamin; Bayer Healthcare, Pymble, NSW 2073, Australia), 0.3 mL IM of Ceftiofur (50 mg/mL; Pfizer, West Ryde, NSW 2114, Australia), and their eyeteeth were clipped and their tails cut and cauterised. At three days after farrowing, all piglets received an oral 1 mL dose of Toltrazuril (50 g/L; Bayer Healthcare, Pymble, NSW 2073, Australia), a repeat 0.3 mL IM of Ceftiofur (50 mg/mL), and 2 mL IM of a *Mycoplasma hypopneumoniae* vaccination (Pfizer, West Ryde, NSW 2114, Australia).

All litters had unlimited access to drinking water from one nipple drinker per farrowing or weaner pen. All piglets were offered creep feed (15.7 MJ digestible energy (DE)/kg; CP, 220 g/kg; 0.88 g available Lysine (av. Lys)/MJ DE; Lienerts Australia 700 Creep Pellet, Roseworthy, South Australia) ad libitum from a rotary feeder with hopper (one feeder per farrowing crate) from 10 days of age before weaning and from a 3-hole weaner hopper (one feeder per weaner pen) after weaning. Sows were fed to appetite with a standard lactation diet (14.1 MJ DE/kg; CP, 160.9 g/kg; 1.05 g av. Lys/MJ DE) during the 28 or 35 days lactation period. Sows had ad libitum access to water through one nipple drinker per farrowing pen.

### 2.2. Treatments

Within each batch, sows were allocated to one of three weaning regimens: (1) conventional weaning (CW28) (*n* = 22); (2) IS with a conventional weaning age (IS28) (*n* = 21); and (3) IS with an older weaning age (IS35) (*n* = 21). The number of litters per treatment per batch ranged from three to four litters. Litters from each of the three treatment groups within a batch were housed in the same farrowing room. Within each weaning regime, the beginning of the experimental procedure (day 0) was designated as the day on which most of the litters were born within that treatment.

The CW28 litters had continuous access to the sow during the 4-week lactation period (day 0 to day 28). In the IS treatments, sows were separated from the litter from 08:00 to 16:00 h daily in the week before weaning (day 21 to day 28 and day 28 to day 35 for IS28 and IS35, respectively). During separation, sows were exposed to a boar for 20 min twice daily in a detection mating area (as part of another experiment measuring ovulation rates; Chen et al. [16]) and then housed individually in a separate building. Within each litter, at day 14, five piglets closest to the average body weight of that litter were selected and individually ear-tagged. Of the piglets used, 52% were female and 48% were males. At weaning, each litter was removed from the farrowing pen. The five individually tagged piglets within each litter remained together and were moved to pens (2.09 m × 1.35 m, consisting of 1.26 m^2^ slatted flooring and 1.56 m^2^ solid flooring), while the rest of the litter was returned to the herd. After day 46, the five individually tagged piglets from each litter were also returned to the herd.

### 2.3. Measurements

Piglets were individually weighed at days 3, 14 (batches 4, 5 and 6 only), 20, 23 (CW28 and IS28 only), 27, 30, 34, 37 (IS35 only), 41, 46 and at 90 days of age. The weights of the individually tagged piglets were recorded to ensure that the performance of the litter before weaning corresponded to the subset of piglets that were followed after weaning. Starting at day 20 (CW28 and IS28) and day 27 of lactation (IS35), creep feed residues were measured on the same days as piglet weights and average daily feed intake (ADFI) was calculated. No food wastage was observed due to the design of the rotary feeder; therefore, disappeared creep feed was considered eaten.

A subsample of piglets from sows in three of the batches (a total of nine piglets per treatment) were subjected to sugar absorption tests (SAT) as markers of intestinal absorption based on methodologies described by Thymann et al. [17], Cox et al. [18] and Berkeveld et al. [19]. Briefly, two piglets were selected per litter and sugar absorption across the GIT using either 10% mannitol (≥98%; Sigma Aldrich, St Louis, MO, USA) (5 mL/kg body weight (BW)) or 10% galactose (≥99%; Sigma Aldrich, St Louis, MO, USA) (5 mL/kg BW) was assessed. The mannitol SAT was a longitudinal study performed 3 days before (4 days after the start of IS for IS28 and IS35 piglets) and 4 days after weaning. A different piglet was used for the galactose SAT, which was performed 4 days after weaning. Piglets in both tests were fasted for 3 h by separation from the sow and removal of creep feed (pre-weaning SAT) or by removal of solid feed (post-weaning SAT), but had free access to water. Piglets from the CW28 group, selected for fasting during the pre-weaning phase, were housed with three other piglets from their litter in pens in a separate room to the experiment litters. To keep conditions consistent, selected piglets from the IS28 and IS35 groups were also housed in pens in a separate room with three other piglets from their litter during the fasting period. An oral dose containing the sugar solution was then administered via a nasogastric tube. Twenty minutes after administration, a blood sample was taken by venipuncture of the jugular vein. The sample was collected in a lithium-heparin coated and an ethylenediaminetetraacetic acid (EDTA)-coated tube and immediately chilled on ice. The blood was centrifuged for 20 min at 2800× *g* at 4 °C and aliquots of plasma were stored at –80 °C.

### 2.4. Determination of Plasma Mannitol and Galactose Concentrations, Cytokine Concentrations, and Cortisol and CRH Concentrations

Plasma mannitol and galactose concentrations were determined using commercial kits (Abcam, ab 155890 D-Mannitol colorimetric assay kit; ab83382 Galactose assay kit, Waterloo, NSW, Australia) in accordance with the manufacturers’ instructions. Plasma from piglets dosed orally with mannitol was analysed for interferon (IFN)-γ, interleukin (IL)-1α, IL-1β, IL-6, IL-10 and tumor necrosis factor (TNF)-α using a multiplex cytokine assay system according to the manufacturers’ instructions (product code MPPCYTMAG23 K06; Millipore; Billercia, MA, USA). Samples and standards were read on a multiplex magnetic bead reader (Bio-plex, Bio-Rad 200, Hercules, CA, USA) and results were analysed using Bio-plex manager’s software (version 5, Bio-Rad, Hercules, CA, USA).

Plasma from the piglets dosed orally with mannitol was also analysed for cortisol and CRH. Plasma cortisol and CRH were determined using commercially available ELISA kits (Enzo Life Sciences, Cortisol ELISA kit, AD-901-071, Farmingdale, NY, USA and BioSource Porcine Corticotrophin Releasing Hormone ELISA kit, MBS267253, San Diego, CA, USA) in accordance with the manufacturers’ instructions with the exception of the optical density for cortisol, which was read at 415 nm instead of the recommended 405 nm. The intra-assay coefficient of variation (CV) for cortisol was 10.5% (low), 6.6% (medium) and 7.3% (high), and ≤8% for CRH. Samples for cortisol and CRH were diluted to 1:25 and 1:40, respectively.

### 2.5. Statistical Analysis and Presentation of Data

Data are presented as means with overall standard error mean (SEM). Performance data were analysed using the generalized linear model (GLM) procedures of SAS (SAS Inst. Inc., Cary, NC, USA) with the litter as the experimental unit. Data for BW, average daily gain (ADG) and ADFI are expressed on a per piglet basis. Body weights were averaged per litter for each day of measurement. Day 3 weights influenced the values for ADG for the period before treatment intervention, eight and five days before weaning, as well as the weaning weights (taken on day 27 for CW28 and IS28 and day 34 for IS35) and the weights on the last day of the experiment (day 46) (*p* < 0.01). Therefore, effects on ADG were always corrected for BW at day 3 for the above-mentioned time points. Results from the GLM for litter ADG between 5 days and 1 day before weaning yielded the same results as that of the subsample of piglets selected for weaning. Data on BW, ADG and ADFI were normally distributed and analysed using the following model:

Y_ij_ = μ + TRT_i_ + bw3 + e_j_, where μ is the overall mean, TRT_i_ is the treatment, bw3 is the body weight at 3 days of age and e_j_ is the residual error.

The growth check experienced over the weaning period was calculated according to a previous report [20] (ADG _2 to 6 days after weaning_ − ADG _5 to 1 days before weaning_).

Plasma mannitol, cortisol, CRH and cytokine concentrations were analysed on a per pig basis (one piglet per litter) using the GLM procedures of SPSS (IBM Corp, Version 21, Armonk, NY, USA) using the following model:

Y_ij_ = μ + TRT_i_ + e_j_, where μ is the overall mean, TRT_i_ is the treatment, and e_j_ is the residual error. Whenever there was an overall treatment effect, differences between two treatments were tested using post hoc Tukey test, maintaining overall Type II error, and litter as the experimental unit. A GLM was also used to compare differences between days within treatment.

Data for galactose and IL-10 were negatively skewed, requiring transformation to force normality using a log_10_ transformation before analysis. The mean values and confidence intervals were then back-transformed and expressed as least square means with 95% confidence intervals. All correlations were investigated using the Pearson’s correlation coefficient. Preliminary analyses were performed to ensure no violation of the assumptions of normality and linearity. Interpretation of batch variation was not reliable due to the small numbers of litters per treatment per batch and therefore batch was not included in any of the statistical models. Statistical significance was accepted at *p* ≤ 0.05 and a trend was considered at *p ≤* 0.1 and *p* > 0.05.

## 3. Results

### 3.1. Production Measurements

**Figure 1:**
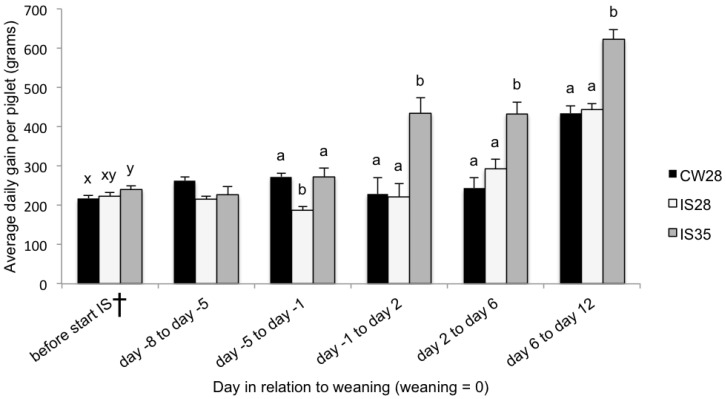
The average daily gain (ADG) of piglets from birth (day three) to 12 days after weaning in conventional weaning and intermittent suckling with or without an older weaning age. The figure depicts the results of three different weaning regimes (CW28 = conventional weaning (*n =* 22); IS28 = intermittent suckling starting at day 28 (*n* = 21); and IS35 = intermittent suckling starting at day 28 (*n =* 21). ^ab^ Values on a specific day not having the same superscript are significantly different. ^xy^ values on a specific day not having the same superscript are trends. † Before the start of the intermittent suckling (IS) treatment intervention for IS28 and IS35.

There was no difference in piglet BW between the three groups from 3 to 46 days of age (Table 1). At 90 days of age there was also no difference in average BW between the groups, resulting in a similar ADG from birth to this time (an average of 0.6 kg per day for all treatment groups, data not shown). Before any treatment intervention, there was a trend for CW28 to have a lower ADG than the IS35 group (Figure 1; *p* < 0.1). However, between five and eight days before weaning, after the start of IS, there was no treatment effect (Figure 1; *p* > 0.05) for ADG. Compared to CW28, IS reduced the growth rate prior to weaning in the IS28 group (Figure 1; *p* < 0.01) but not in the IS35 group. Weaning was associated with a growth check in the CW28 group (−34 g/day); however, in both IS groups, there was no such growth check (105 g/day and 160 g/day, for IS28 and IS35, respectively). Between one day before weaning to two days after weaning, two days after weaning to six days after weaning, and 6 days after weaning to 12 days after weaning, the IS35 group maintained a higher ADG (*p* < 0.01) than the IS28 and CW28 groups (Figure 1).

### 3.2. Piglet Feed Intake

Two days after the start of IS (between five and eight days before weaning), the IS35 treatment group were eating more than twice the amount of the CW28 and IS28 groups (Table 2; *p* < 0.05). While the changes in feed intake from one and five days before weaning remained negligible for CW28 and IS28, the feed intake of IS35 piglets continued to increase, remaining higher than CW28 and IS28 (Table 2; *p* < 0.001). In the peri-weaning period (one day before weaning to two days after weaning), the CW28 and IS28 groups more than doubled their feed intake compared with pre-weaning values. There was no treatment effect (*p* > 0.05) between CW28 and IS28 groups during the post-weaning period; however, the IS35 group continued to have the highest feed intake (*p* < 0.001) in the 12 day after weaning (Table 2).

When data from all treatment groups were combined, ADFI measured between five and eight days before weaning was highly correlated to ADFI between one to five days before weaning (*r* = 0.77, *p* < 0.001) and feed intake from one day before weaning to two days after weaning (*r* = 0.72, *p* < 0.001). However, the strongest correlation was seen between ADFI from one to five days before weaning and one day before weaning to two days after weaning (*r* = 0.80, *p* < 0.001). A weaker positive correlation also existed between growth rate at one day before weaning (between one to five days before weaning) and feed intake at two days after weaning (between one day before weaning and two days after weaning) (*r* = 0.55, *p* < 0.001).

### 3.3. Sugar Absorption Tests

Mannitol concentrations did not differ (*p* > 0.05) between treatments at three days before or four days after weaning (Table 3). When data from the IS groups were combined and compared to CW28, there was a tendency for the CW28 group to have higher mannitol concentrations than IS at three days before weaning (1250 ± 220.8 nmol/mL versus 811 ± 156.1, respectively; *p* < 0.1). The CW28 piglets had the biggest decrease in mannitol concentration when comparing pre-weaning to post-weaning values within treatment (*p* = 0.01). The IS28 group showed a trending decrease in mannitol concentrations from before weaning to after weaning (*p* = 0.1), and there was no difference in IS35 piglets for pre-weaning versus post-weaning mannitol levels. When data from all three treatment groups were combined, values for mannitol concentrations before weaning were higher than values after weaning (957 ± 131.4 nmol/mL versus 431 ± 41.5 nmol/mL, respectively; *p* < 0.01). Four days after weaning, the IS35 group had the highest galactose concentrations (Table 3; *p* < 0.01).

### 3.4. Cytokine Concentrations

A high proportion of the samples for IFN-γ, IL-α, IL-1β and IL-6 fell below the detection limit of the respective standard curve, meaning that the remaining sample size was insufficient for statistical analysis. However, a sufficient number of samples read in the range of the standard curve for IL-10 and TNF-α. Weaning had an effect on IL-10 concentrations within the CW28 and IS28 treatment groups, with higher post-weaning values compared with pre-weaning values (137 (66.4–282.5) versus 335 (186.6–599.8) pg/mL for CW28, *p* < 0.05; and 153 (71.9–326.6) versus 258 (147.9–450.8) pg/mL for IS28, *p* < 0.05). Although the post-weaning IL-10 concentration for IS35 piglets was numerically higher than the pre-weaning value (126 (54.5−290.4) versus 288 (160.7–516.4) pg/mL), it was not statistically significant. Trends within the IS35 treatment group between pre-weaning and post-weaning values were evident with TNF-α concentrations increasing after weaning (25 ± 10.4 pg/mL versus 44 ± 29.1 pg/mL, *p* < 0.1).

### 3.5. Cortisol and CRH Concentrations

The CW28 group had the highest (*p <* 0.05) cortisol concentration before weaning compared with IS35, and tended (*p* < 0.1) to have a higher cortisol concentration than IS28 (Table 4). This effect disappeared by four days after weaning. The IS35 group had the highest plasma CRH concentration three days before weaning compared with the other treatment groups (*p* < 0.05), but there was no treatment effect for CRH at four days after weaning. Overall, when data from all treatments were combined, the pre-weaning cortisol values were higher than the post-weaning values (118 versus 75 ng/mL; *p* < 0.05). There was no significant difference between pre-weaning and post-weaning values for CRH (Table 4). When comparing pre-weaning and post-weaning values within treatment, the CW28 and IS28 groups both had a trending decrease in cortisol from before to after weaning (*p* = 0.10 and *p <* 0.1, respectively), and there was no change for the IS35 group (*p* > 0.05). There was no change (*p* > 0.05) in CRH concentration within any of the treatment groups over the weaning period.

## 4. Discussion

The aim of this study was to determine if IS with or without an older weaning age provides an adaptation advantage for the GIT during the pre-weaning period, possibly through a reduction in the stress and inflammatory responses, resulting in improved post-weaning piglet performance and GIT function. Results from the current study showed that IS for one week before weaning at day 28 made no difference to the absorption of mannitol and galactose across the GIT compared with a conventional weaning regime. There was also no difference in the cytokine response, and, in contrast to results from previous studies [9,12,15], post-weaning growth and solid feed intake was not increased by IS. Intermittent suckling for one week in combination with an older weaning age (day 35), however, improved feed intake in the 8 days before and the 12 days after weaning, improved growth rate within 12 days of weaning, and improved galactose absorption at 4 days after weaning. There was no effect on the cytokine response. A CW group with an older weaning (day 35) could not be included in this study, and, therefore, comparing measured parameters of IS35 piglets with that of CW28 and IS28 piglets implicates the effects of both IS and an older weaning age.

During lactation, piglets subjected to IS between day 21 and 28 of age experienced a reduction in growth after the onset of IS which lasted until one day before weaning. This pattern is consistent with results from other studies [9,12,15], with Berkeveld et al. [15] suggesting that creep feed intake was insufficient to compensate for the loss of nutrient and energy intake from the reduction in milk consumption due to the separation. Despite a reduction in growth during lactation in the same IS studies, a 12 h/day separation for 11 days before weaning still stimulated solid feed intake before and after weaning [9,12], whereas a 10 h/day separation for 7 days did not stimulate solid feed intake before weaning, but solid feed intake was higher than conventionally weaned pigs for 2 days after weaning [15]. In the current study, an 8 h/day separation for 7 days before weaning neither stimulated solid feed intake before or after weaning compared with conventionally weaned pigs. The difference in results, especially with regard to post-weaning performance, could be due to the fact that primiparous progeny were used, although a study by Turpin et al. [21] showed primiparous piglets separated for 8 h per day 7 days before weaning ate more creep feed before weaning and had a tendency to grow faster than conventionally weaned pigs after weaning. It is also possible that CW28 piglets in the current study may have been exposed to less severe stressors at weaning than under commercial conditions allowing them to perform better than expected. The current study was performed at a research facility where piglets were not mixed or transported at weaning. The housing of piglets with littermates after weaning is associated with less aggression [22] and reduced elevations in cortisol [23]. In this regard, the effects of weaning on CW28 piglet performance would likely be exacerbated under commercial conditions.

In studies by Berkeveld et al. [20] and Berkeveld et al. [15], an IS period lasting two weeks or more caused a marked reduction of the post-weaning growth check. Since ADG measured in the first experimental period after weaning was between one day before weaning to two days after weaning and not from the day of weaning in the current study, it is difficult to draw firm conclusions regarding the growth check since it is not uncommon for conventionally weaned piglets to grow more than 200 g/day just before weaning with zero, or indeed negative growth, immediately after weaning [24]. However, CW28 pigs did experience a reduction in growth during the week after weaning compared with the week before weaning, whereas IS28 pigs experienced a reduction in growth during the week of IS with no growth check over the weaning period. In this regard, it seems that IS is associated with similar problems to abrupt weaning, shifting part of the problems associated with weaning into the lactation period [15]. In saying this, however, given IS piglets did not experience a growth check over the weaning period, one could assume that IS piglets were more familiar with the weaning process and were perhaps less stressed, although the neuroendocrine data from this experiment does not support this assumption, possibly due to sampling time and additional procedures. When IS is combined with an extended lactation, however, recovery is much quicker as evidenced by the IS35 group’s ADG results, but caution must be used to attribute the beneficial effects of the longer IS treatment, since an older weaning age per se has also been shown to improve post-weaning performance [7]. In the current study, due to the lack of a non-IS group weaned at 35 days, the effect of age and IS in the IS35 group performance cannot be separated.

An important aspect of a modified weaning regime, such as IS, is its potential impact on the GIT, since weaning causes marked changes in GIT structure and function, as evidenced by altered nutrient and electrolyte transport, villous atrophy, increased mucosal permeability and inflammation [3]. The SI absorptive capacity of two sugars, mannitol and galactose, was used as an indicator of overall GIT function in the current study. The observed pattern of mannitol absorption in the CW28 piglets corresponded to previously reported values [19]. Although some mannitol passes through the epithelial layer via the paracellular route, it is mostly absorbed via the transcellular route via water-filled pores in the enterocyte membrane, making mannitol a surrogate marker of functional mucosal mass in the SI [18]. In the pre-weaning period, the trend for the IS groups (when combined) to have lower mannitol concentrations than the CW28 group suggests that IS reduces intestinal surface area before weaning. Since solid feed intake at the time of the pre-weaning SAT was similar across treatments and no inflammatory response was evident in the measured cytokines, it is possible that the reduction in intestinal surface area may be a normal physiological adaptive response made more evident by the gradual nature of the IS weaning technique [25]. This is also highlighted by the nearly three-fold decrease in mannitol in the CW28 group over the peri-weaning period compared to an approximate two-fold decrease in the IS groups, suggesting more sudden changes to GIT morphology in CW28 piglets. The lack of treatment effect after weaning is consistent with results from a study by Berkeveld et al. [15], in which no differences in villous height were observed between piglets weaned at day 26 with or without a week of IS. However, in the same study, when lactation was extended to day 33, villous height was higher eight days after weaning, suggesting that improvements in SI morphology seemed to be an effect of age and possibly secondary to a higher feed intake. However, the current study found no difference in IS35 mannitol concentrations compared to the other treatment groups after weaning, which may be a result of timing of the measurement (four versus eight days after weaning in the Berkeveld et al. [15] study) and/or a result of the limitation of mannitol as a measure of intestinal surface area.

In contrast, the post-weaning absorption of galactose was improved in the IS35 group compared to the other treatment groups. The absorption of galactose is similar to glucose via a sodium-glucose linked transporter (SGLT1), which relies on the function of the Na^+^-K^+^ ATP pump to create a Na^+^ gradient across the cell membrane to function [26]. The change from a milk-based feed to a solid feed increases Na^+^-K^+^ ATP pump activity [27], therefore enhancing glucose and galactose absorption. In the current study, the greater solid feed intake in the IS35 group four days after weaning may have had the same effect on Na^+^-K^+^ ATP pump activity and resulted in a higher absorption of galactose.

Cytokines are essential for the activation of neutrophils, macrophages, T cells, B cells and dendritic cells to protect the integrity of the epithelium, and also play a role in body defense [28]. The predominant effect of IL-10 is to reduce inflammation; therefore, the upregulation of IL-10 after weaning is consistent with the inflammatory response seen around weaning [29]; however, there are no weaning studies to our knowledge that have examined the IL-10 response. The values for TNF-α over the weaning period in the IS35 group are much lower than other studies where weaned pigs were subjected to an immune stressor (4000 pg/mL for the stressed pigs) and resemble values reported in controls in the same experiment (50 pg/mL) [30]. Therefore, the results in the current study are not likely to be associated with an inflammatory response. The lack of response in the other cytokines measured may have been the result of low sample numbers, the low stress research environment (mentioned previously) or the timing of when the sample was taken (four days post-weaning). Pié et al. [31] reported increases in cytokine gene expression along the GIT one to two days after weaning. The late sampling may also explain the post-weaning increase in an anti-inflammatory cytokine (IL-10) rather than pro-inflammatory cytokines.

In the current study, blood samples to measure cytokines, cortisol and CRH were taken from piglets that had fasted for 3 h and underwent nasogastric intubation to receive an oral dose of mannitol or galactose. Although these procedures did not appear to impact the cytokine response, when interpreting the cortisol data, one needs to consider that CW28 piglets had to be separated from their dams during the pre-weaning fasting period. Psychosocial stress in the form of maternal separation during early postnatal life has been shown to induce deleterious changes in neuroendocrine functions and behaviour in many different species [32,33,34]. The higher levels of cortisol seen in the CW28 piglets before weaning during their first experience of maternal separation compared with piglets that had been subjected to maternal separation for three days (IS28 and IS35 groups) were consistent with results from a previous study from the same research group, where IS piglets experienced a transient increase in cortisol on the first day of separation but were attenuated by the last day of separation [21]. Furthermore, the plasma cortisol values in the current study were generally much higher than one would expect from weaning associated stress [29,35]. Plasma cortisol concentrations ranging from 60–108 ng/mL have previously been associated with procedures such as electrical stimulation, negative handling and the use of a snout rope [36]. Since previous weaning studies only involved venipuncture of the piglets at weaning, it is most likely that the nasogastric intubation strongly influenced the cortisol values in the current study. In saying this, however, all treatment groups were treated the same way before and after weaning, making comparison between the groups and time points possible. Overall, pre-weaning cortisol values across all treatments were higher than the post-weaning values. This was unexpected given reported results from most other experiments measuring plasma or urinary cortisol concentrations over the weaning period showed an increase in cortisol after weaning [4,37,38,39]. Alternatively, there are some studies that failed to find any increase in cortisol concentrations associated with weaning despite changes in other measures including growth, behavior and hormones [40,41]. It is possible that the choice of sample time meant that the acute post-weaning rise in cortisol was missed or intubation with a nasogastric tube the first time (before weaning) could have been more stressful than the second time (after weaning).

The number of studies examining plasma CRH in the peri-weaning period are limited, however, results generally have shown that CRH follows the same pattern as cortisol [4,42] but with changes in CRH lasting for a shorter period of time after weaning. This is presumably due to the negative feedback response exerted when glucocorticoid concentrations are high [43]. The reason why the IS35 group had higher pre-weaning plasma CRH concentrations compared with the other treatment groups is not known. Once again, the process of nasogastric intubation most likely influenced the CRH values, resulting in much higher concentrations than one would expect for weaning-associated stress [4,29,42]. However, this difference could also be explained by the effect of sedation with piglets in the current study being fully conscious during venipuncture, whereas piglets in the previously mentioned weaning studies were sedated. It has previously been mentioned that CRH may be a more sensitive stress indicator of GIT barrier dysfunction [4]; however, the authors are unable to comment on the effect that high CRH values may have had on the intestinal mucosa of piglets in the current study, since GIT permeability was not measured. Overall, the lack of treatment effect in the CRH values between CW28 and IS28 before weaning suggests that short periods of maternal separation (8 h/day) do not affect the piglet stress response before or after weaning.

## 5. Conclusions

Overall, the combination of IS with an older weaning age (day 35) increased solid feed intake before and after weaning resulting in higher growth rates 12 days after weaning and an improvement in GIT absorptive function (as assessed by galactose absorption). However, and due to the lack of non-IS group weaned at 35 days, the effect of age and IS in the IS35 group on performance cannot be separated. Differences in ADG had disappeared by the time pigs reached 90 days of age. Unfortunately, since cytokine and neuroendocrine data were potentially influenced by procedures, such as the oral dosing of sugars, it is difficult to draw firm and unifying conclusions about the effect IS has on the neuroendocrine and inflammatory responses. Nevertheless, the minimal differences between the treatments suggest that, apart from an initial peak in cortisol, short periods of maternal separation (8 h/day) do not affect the piglet stress response before or after weaning, but neither do they add any adaptation advantage to the process of weaning. In conclusion, IS involving an 8 h daily separation the week before weaning does not appear to prevent weaning-associated changes, but rather advances them in an attenuated way. This provided a more gradual transition into weaning and was seemingly more pronounced when piglets were weaned at a later age (i.e., 35 days).

## Figures and Tables

**Table 1:** Effect of intermittent suckling with and without older weaning age on the body weight of piglets between 3 and 90 days of age.

	Treatment ^1^			SEM	*p* Value
	CW28	IS28	IS35		
BW, kg					
Day ^2^					
3	1.8	1.6	1.6	0.08	0.27
14 ^3^	3.7	3.7	3.7	0.24	0.97
20	5.5	5.5	5.0	0.23	0.30
27	7.4	6.8	6.9	0.23	0.19
30	8.1 *	7.5 *	7.6	0.24	0.20
34	9.0	8.7	8.7	0.27	0.55
37	-	-	10.0 *	0.36	-
41	12.1	11.8	11.7	0.35	0.72
46	14.8	14.5	14.5	0.43	0.83
90	75.2	77.1	74.7	1.51	0.50

^1^ CW28 = Conventional weaning at day 28; (*n* = 22); IS28 = Intermittent suckling starting at day 21 (*n* = 21); IS35 = Intermittent suckling starting at day 28 and weaning at day 35 (*n* = 21). ^2^ Day of the experiment (day 0 = birth of piglets). ^3^ 11 litters per treatment for day 14 only (only batches 4, 5 and 6 were weighed). * Indicates the first weighing after weaning. BW: body weight; SEM: standard error mean.

**Table 2:** Effect of intermittent suckling with and without an older weaning age on the average daily feed intake of piglets before and after weaning.

	Treatment ^1^			SEM	*p* Value
	CW28	IS28	IS35		
ADFI (g)					
Days before weaning					
−8 to day −5 ^2^	35 ^a^	30 ^a^	75 ^b^	8.3	<0.05
−5 to day −1	43^a^	33 ^a^	114 ^b^	9.1	<0.001
Days after weaning					
−1 ^3^ to day 2	111 ^a^	106 ^a^	255 ^b^	14.9	<0.001
2 to day 6	274 ^a^	290 ^a^	435 ^b^	23.3	<0.001
6 to day 12	459 ^a^	511 ^a^	670 ^b^	25.6	<0.001

^1^ CW28 = Conventional weaning (*n* = 22); IS28 = Intermittent suckling starting at day 21 (*n* = 21); IS35 = Intermittent suckling starting at day 28 (*n* = 21). ^2^ Day in relation to weaning; the minus symbol is the day of measurement prior to weaning (e.g., −8 is eight days before weaning).^3^ Creep feed intake was measured the day before weaning. ^a,b^ Values within a row not having the same superscript are significantly different. ADFI: average daily feed intake.

**Table 3:** Effect of intermittent suckling with and without an older weaning age on plasma mannitol and galactose concentrations (nmol/mL) of piglets before and after weaning.

		Treatment ^1^			SEM	*p* Value
		CW28	IS28	IS35		
	Day ^2^					
Mannitol (nmol/mL)	−3	1250	874	747	224.6	0.28
	4	471	411	417	79.7	0.83
Galactose ^3^ (nmol/mL)	4	70 ^a^ (34.3–143.9)	133 ^ax^ (64.9–272.3)	316 ^by^ (135.2–751.6)		0.03

^1^ CW28 = Conventional weaning (*n* = 9); IS28 = Intermittent suckling starting at d 21 (*n* = 9); IS35 = Intermittent suckling starting at d 28 (*n* = 9). ^2^ Day in relation to weaning, the minus symbol is the day of measurement prior to weaning (e.g., −3 is three days before weaning). ^3^ Data were logarithmically transformed before being subjected to the generalized linear model (GLM). Values were then back-transformed and expressed as least square means with 95% confidence intervals (in parenthesis). ^a,b^ Values within a row not having the same superscript are significantly different. ^x,y^ Values within a row with different superscripts are trends.

**Table 4:** Effect of intermittent suckling with and without an older weaning age on cortisol and corticotrophin releasing hormone (CRH) concentrations (ng/mL) of piglets before and after weaning.

		Treatment ^1^			SEM	*p* Value
		CW28	IS28	IS35		
	Day ^2^					
Cortisol (ng/mL)	−3	160 ^ax^	110 ^ay^	83 ^b^	19.5	0.03
	4	96	60	72	23.5	0.55
CRH (ng/mL)	−3	10 ^a^	9 ^a^	13 ^b^	1.1	0.03
	4	10	10	12	1.0	0.21

^1^ CW28 = Conventional weaning (*n* = 9); IS28 = Intermittent suckling starting at day 21 (*n* = 9); IS35 = Intermittent suckling starting at day 28 (*n* = 9). ^2^ Day in relation to weaning, the minus symbol is the day of measurement prior to weaning (e.g., −3 is three days before weaning). ^a,b^ Values within a row not having the same superscript are significantly different. ^x,y^ Values within a row with different superscripts are trends.
